# Knowledge assessment of trainees and trainers in general practice in a neighboring country. Making a case for international collaboration

**DOI:** 10.1186/1471-2296-13-103

**Published:** 2012-10-15

**Authors:** Roy Remmen, Johan Wens, Annelies Damen, Herman Duesman, Veronique Verhoeven

**Affiliations:** 1 Department of Primary and Interdisciplinary Care, Faculty of Medicine and Health Sciences, University of Antwerp, Campus Drie Eiken, Building R, room 3.27, Antwerp, Belgium; 2 Interuniversity Center for Training in General Practice, Leuven, Belgium; 3 Institute for Education Training and Evaluation in General Practice, Utrecht, The Netherlands

**Keywords:** General practice/family medicine, Quality, Postgraduate training, Guidelines, International

## Abstract

**Background:**

In Europe, a comparable scope of training in GP can be observed especially in the field of knowledge. This feasibility study determines if a knowledge test is suitable in the context of a neighboring country.

**Methods:**

A Dutch knowledge multiple choice test was used after validation of its content in Flanders (Belgium) in the academic year 2010–2011. Satisfaction with the test format was assessed. The test was taken by general practice trainees and trainers. Group scores of trainees in year 1, 2 and 3 and their trainers were compared to Dutch participants as a control group.

**Results:**

80 percent of the items in the Dutch test were transferable to Flanders (Belgium). Flemish participants (Belgium) liked the test format. The scores of all Belgian participants groups were lower than the Dutch participants.

**Conclusion:**

The results among 1278 participants show that the use of the Dutch knowledge multiple-choice test is feasible in a neighboring country. At present, the individual scores can not be used for high stake decisions for trainees in Flanders (Belgium). If countries collaborate in the area of assessing GPs trainees, there would be an economical benefit due to increased efficiency. It would also lead to greater international integration of the discipline.

## Background

The European Definition of General Practice/Family Medicine describes the domains that should be covered by general practitioner training (GPs) [1]. One of the key determinants of successful training in General Practice is the ability to use specific specialist knowledge.

Assessing knowledge is difficult. There is evidence that simple multiple choice testing correlates well with other, far more expensive test formats [2]. In the undergraduate curriculum there is evidence suggesting that core knowledge can be captured in multiple choice tests assessment format [3]. Multiple choice tests were capable of identifying trends between students in different years, and also between participating medical schools [4,5]. Designing good knowledge tests is however, a resource-intensive task. Indeed, the amount of time and effort taken to produce test items of acceptable quality can be underestimated. Sharing test material across institutions provides an economic benefit that would allow a more quality driven process for test construction [6]. There is some evidence that collaboration is also feasible in an international setting. For instance, Remmen et al. concluded that between two bordering countries, differences between groups of students could be shown. Students at 4 universities in two countries scored significantly different and this was attributed to the curriculum designs [7].

Training for general practice (GP) in Europe is defined by a directive, indicating its content [1]. General Practice training in Europe, even within different health systems shows an increasing comparable scope especially in the field of knowledge, so the need for more uniform assessment within the European context is apparent. The need to investigate this further was also suggested by the research agenda for General Practice in Europe, to explore instruments to assess students in the discipline [1].

At present, medical specialties tend to develop national assessment procedures [8]. In the United Kingdom and Australia for instance, high stake examinations were developed to assess clinical knowledge [9,10]. At least one professional organization (Radiotherapy and Oncology) of medical specialties informally introduced transnational training and assessment procedures [11]. Although these projects do not have legal and high stake consequences, they are innovative as they define and share the core knowledge of a discipline [12].

The evidence base of the knowledge of general practice across many health systems is to a great extent comparable [13]. Given the potential gains in collaboration to develop good assessment material we set out to test the feasibility for cross border cooperation in General Practice. As a prerequisite for further collaboration, an important question is ‘Can test material can be used in another country?’ This report describes the first exploratory study to ascertain whether or not it is feasible to use a Dutch multiple choice knowledge test in a neighboring country.

### Context

The health systems in the two participating countries differ but GPs tend to cross the border easily and the language is almost identical. In the Netherlands there is a long history of guideline preparation and adherence while in Belgium (Flanders) there is great emphasis on accessibility at all levels of service [14,15]. In this respect, Belgium (Flanders) resembles more the approach of southern Europe while the Netherlands shows stronger primary care.

Both countries have a long-standing culture of GP training. In the Netherlands training lasts three years after the undergraduate curriculum. Students tend to have an intermediate period before entering training while in the meantime working as a junior doctor not in training. The eight universities organize postgraduate training, but assessment is centrally organized in a consortium of eight universities.

In Belgium (Flanders) students enter the vocational period in the final year (year 7, the first year of general practice training) of the undergraduate curriculum. Subsequently, they enter training without an intermediate period that is entirely organized by an inter-university consortium (year 2 and 3, so the second and third year of general practice training). The educational efforts of the consortium was awarded the excellence criterion in a joint (Dutch/Belgium (Flanders)) accreditation [16]. The average age of trainees is 27 years in Flanders (Belgium) and 31 years in the Netherlands. This difference is due to slower progress during undergraduate training and waiting lists for GP training in the Netherlands [17]. In both countries about 65 percent are female trainees.

In the Netherlands ‘The National GP knowledge test’ (LHK-test) is obligatory at fixed times through postgraduate training. Students should at least acquire a pass rate once a year [18]. For each test, a blueprint is used to cover all domains using for this chapters of the International Classification of Primary Care (ICPC). Until 2010, an item format with a three answer category: yes, no, don’t know, was used. Recent tests consist of standard multiple choice questions with a short list of alternative solutions. A recent study concluded insufficient knowledge in the general practice field, using this test together with older age of the trainee and insufficient general practice competencies at the start of the GP-training which were considered risk factors for drop-out or insufficient progress during training [19].

At the level of the steering committees of the inter-university centers for GP training in both countries it was decided to pilot the Dutch knowledge test in Belgium (Flanders).

## Method

### Process of implementation

We recorded the process of the implementation of this Dutch assessment format in Flanders from February to May 2011. Acceptance of this test of trainees and teachers is needed for further cooperation. Therefore, at test delivery in Belgium (Flanders), we also included a 4-item satisfaction questionnaire to identify participants’ opinions and we asked how much time was needed to do the test.

### Instrument

In Flanders we used an existing Dutch test consisting of 155 questions, which represents the entire domain of general practice using the Dutch test blueprint based on ICPC domains (see Table 1). In the blueprint, more prevalent GP topics generated more questions. So for instance, the domain Circulatory (16 items) is more prominent than a knowledge area such as Blood domain (2 items) or Eye domain (6 items). The questions are divided into 17 fixed chapters: 16 ICPC-chapters and a GP-theoretical chapter. Questions cover age-dependent complaints, acute and chronic illnesses and typical aspects of a consultation in General Practice. All questions are posed as a case vignette with three possible answers: correct, incorrect or question mark. To allow for guessing, a good minus incorrect score (G-I) was calculated per sub-group and 95% confidence intervals were calculated. For each incorrect answer one point was subtracted. For the Dutch participants, we used the historical data of results of the items in the final instrument to make a comparison with the results of the Belgian participants.

**Table 1:** Subdomains of the blueprint of the knwowledge test

**Subdomain**	**Number of items**	**Percentage**
General (ICPC A)	9	6
Blood system (ICPC B)	2	1
Digestive system (ICPC D)	12	8
Eye (ICPC F)	6	4
Ear (ICPC H)	7	5
Circulatory system (ICPC K)	16	10
Locomotor system (ICPC L)	17	11
Nerve system (ICPC N)	8	5
Mental and psychology (ICPC P)	8	5
Respiratory system (ICPC R)	16	10
Skin (ICPC S)	12	8
Endocrine system (ICPC T)	6	4
Urinary tract (ICPC U)	6	4
Pregnancy and anticonceptive (ICPC W)	7	5
Female genital tract and breasts (ICPC X)	7	5
Male genital tract (ICPC Y)	6	4
General practice and theory	10	6
**Total**	**155**	**100**

For pass/fail decisions there is discussion whether to use a fixed or relative standard in multiple choice testing [20]. The use of a relative standard has the advantage of correcting for variations in test-difficulty. In the Netherlands, therefore, a relative standard is used. A pass is defined as a score equal to the mean score of all participants in a sub-group (i.e. GP trainees in year 1,2 and 3 and GP trainers), minus one standard deviation.

### Validation in Belgium (Flanders)

To check for local face validity two Flemish senior trainers from the University of Antwerp independently analyzed 160 items of the Dutch October 2008 test. Firstly, they individually identified items of this test which did not fit into the Flemish situation. They identified items regarding the health system that were not applicable in Belgium (like legal and ethical issues of the health care system of the Netherlands). After one meeting, 21 questions were omitted from the test in mutual agreement. To assure the test would be robust enough to cover the entire blueprint of ICPC codes, 16 items from the item bank were subsequently included in the LHK-test by mutual agreement between the senior Flemish trainers and the Dutch representative. In the end, the test consisted of 155 items but the original blueprint was retained (see Table 1).

### Participants

We aimed to compare the results of the first to the third year GP-trainees, and the GP trainers in the two countries. Scores on the same items were reproduced from the Dutch database that was collected by the Institute for Education Training and Evaluation in General Practice, The Netherlands. These participants come from all eight Universities and did the test as a formal examination and were used as a reference group. Data of a total number of 887 Dutch participants (744 GP-trainees and 123 GP-trainers) were included in the analysis. Routine assessment data was used.

In Flanders (Belgium), a total of 353 GP-students and 58 GP-trainers from the four Flemish Universities participated in the study and for them the test was not a formal examination. Written informed consent for participation in the study was obtained. All participants received a personal feedback two weeks after doing the test and could opt for their personal data to be eliminated from the database.

In order to pass this voluntary exercise, trainees were instructed that individual scores should be more than the mean score minus one standard deviation. In one subgroup in Flanders (Belgium) students could earn a credit (year 1, Institute Two) if their score was a pass (so the Flemish mean minus one SD). They were asked to study the national guidelines of the discipline.

Ethical Approval for this study was granted (number 11/10/93 of the University Hospital of the University of Antwerp) and this included the use of the routine examination data of the Dutch database. Ethical approval in the Netherlands was not requested because the data were anonymous and from a routine database.

### Statistics

For the statistical analysis we used SPSS version 18. We used descriptive statistics and analysis of variance for comparing groups (GP-students and GP-trainers) and confidence intervals of differences of scores were calculated. To test for normal distribution Kolmogorov Smirnov test was used.

### Process of implementation

During the process of implementation in Flanders we dealt with a number of difficulties. The universities all agreed except one (university Four) to participate with the students of year 1. For year 2 and 3, the agendas of trainees and trainers were nearly saturated and to overcome resistance, efforts were necessary to motivate trainers. We visited their training sessions and discussed goals and process of the project with them. In general, the trainees did not obstruct the process at any time. In the preparation phase we managed to convince the GP-trainers and trainees to participate voluntarily in this test case, partly due to the timing of the test. So we sent all participants a motivational mail a few weeks before the test case. We suggested some potential benefits such as acquiring information about the content of a test in a neighboring country and the possibility of a personal report and insight into personal knowledge gaps. For the GP-trainers 2 credit points for their individual accreditation was given as an incentive.

### Test satisfaction questionnaire

**Figure 1:**
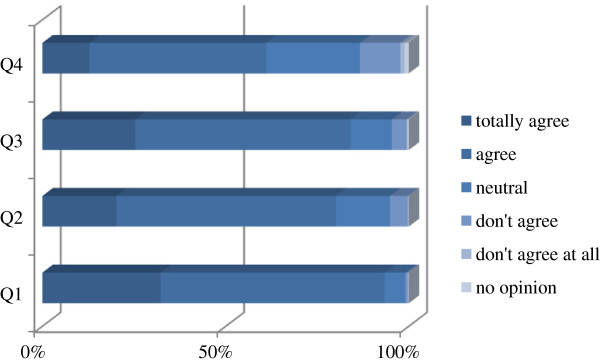
**Answers of Flemish respondents to the satisfaction questionnaire.** Question 1 (Q1): The topics in the test were relevant for general practice. Q2: The questions in the test were relevant for general practice. Q3: The test helps me to determine the gaps in my knowledge. Q4: The test can help me in deciding which refresher courses to take.

The majority of respondents succeeded in completing the test within one to two hours. Figure 1 presents the satisfaction of participants and time needed to do the test.

## Results

The Dutch cohort consisted of 744 GP-trainees and 123 GP-trainers and the Flemish cohort consisted of 353 GP-trainees and 58 GP-trainers (Table 2). Score variances between comparable groups of the same level differed little. Also, normality of score distributions within groups was tested as not significantly different from a normal distribution (Kolmogorov Smirnov test, P < 0,05). For the Flemish sample, the response rate of year 1 was 48.2%, year 2: 93.4%, year 3: 59.7%, and 11.2% of the GP trainers participated. Dutch response rates were 92 percent on average as testing is part of routine examinations. Table 3 shows the global test results of the groups of participants. The table shows that all Dutch groups (year 1, year 2, year 3 and GP-trainers) have higher mean scores than all Flemish groups, and the SD is slightly smaller in the Dutch groups.

**Table 2:** Mean scores (MS) of good minus incorrect scores of groups in two countries

**Country**		**Flanders, Belgium**			**The Netherlands**	
**Group**	**N**	**MS**	**SD**	**N**	**MS**	**SD**
year 1	110	40,0	10,2	259	47,2	9,9
year 2	157	38,5	10,0	262	48,6	9,8
year 3	86	43,3	9,2	223	51,0	9,1
Trainers	58	38,4	11,1	123	50,3	10,6

(*SD*) Standard Deviation, (*N*) N number of participants.

**Table 3:** Mean scores of good minus incorrect scores in Belgium (Flanders)

**University**		**One**			**Two**			**Three**			**Four**	
**Group**	**N**	**MS**	**SD**	**N**	**MS**	**SD**	**N**	**MS**	**SD**	**N**	**MS**	**SD**
year 1	26	40,4	10,7	23	47,2	7,7	61	37,2	9,6	-	-	-
year 2	88	35,6	9,3	13	42,3	9,4	46	44,2	8,2	10	32,1	10,9
year 3	44	41,1	8,8	9	43,5	5,7	28	46,3	10,3	5	45,6	7,9
all	158	38,0	9,7	45	45,0	8,0	135	41,5	10,1	15	36,6	11,8

(*SD*) Standard Deviation, (*N*) N number of participants.

In the Flemish test groups there is no significant difference in the good minus incorrect score (G-I score) between year 1 participants (mean G-I score: 40, SD: 10.2), year 2 participants (mean G-I score: 38.5, SD: 10) and GP-trainers (mean G-I score: 37.4, SD: 11.1). Year 3 scores (mean G-I score: 43.3, SD: 9.2) were significantly better than year 1 (p=0.02, 95% confidence interval 0.53-6.12), year 2 (p=0.00 95% confidence interval 2.28-7.49) and the scores of the GP-trainers.

Table 3 also reports the results of participating subgroups in Flanders. When we compare the good minus incorrect score of GP students (n=353) to that of GP-trainers (n = 58) using analysis of variance, there is no significant difference (p=0.061, 95% confidence interval 1.45-5.55). There was only a significant difference in G-I score between year 3 (n=86, p=0.01, 95% confidence interval 1.70-2.56) and GP-trainers. No significant difference was found between year 7 (n=110, p=0.130, 95% confidence interval 0.78-1.71 and GP-trainers and between year 8 (n=157, p=0.514, 95% confidence interval 1.58-2.09) and GP-trainers.

We noticed differences between the universities (Table 3). For instance in Belgium, University Two scored significant better for the G-I score than University One (p=0.00, 95% confidence interval 0.84-10.33), University Three (p= 0.034, 95% confidence interval 0.28-6.88) and University Four (p=0.004, 95% confidence interval 2.75-14.19). University Three scored significant better for the G-I score than University One (p=0.002, 95% confidence interval 1.26-5.75).

## Discussion

Our results show that the use of the Dutch knowledge multiple choice test is feasible in a neighboring country. Firstly, more than 80 percent of the Dutch items were considered appropriate in Flanders. This suggests that the core of the knowledge base of general practice is much the same in these two countries. Secondly, although we had to overcome some resistance among the GP trainers, the great majority of Flemish participants liked the test and thought the items of the test represent core knowledge of the discipline. The major critique was that some questions were posed in “too Dutch” language for the Flemish participants. This suggests that the review of the items was performed by senior clinicians who understood the Dutch wording, whereas the Belgium (Flanders) students did not grasp the entire meaning of some words in the items. Although the citizens of Flanders and the Netherlands speak almost the same language, in future we need to attach importance of sensitivity for the technical idioms of each language. When expanding efforts to countries with another language this issue needs even more consideration but evidence from the undergraduate curriculum suggests that in the majority of cases only a small amount of work is needed to adapt questions [21].

Can this bilateral collaboration be sustained? At present, the Dutch test is being introduced as a formative instrument and will be offered to all trainees and teachers in general practice in Belgium (Flanders). More experience with the procedures and results will probably help to overcome initial resistance to 'foreign' test formats. There needs to be more input from the Flemish teachers in the process of item banking in future cooperation. This academic commitment would help to establish a sense of ownership. Although Dutch is the language in both countries, some idioms are not readily transferable and this needs attention in the future. As Dutch is a minority language, the discussion of language should, in the broader European context, also deal with the possibility to select the English language, but this would also pose similar problems in the use of idioms.

We used aggregated group scores and we did not report scores per ICPC domain because the number of participants was too small to give meaningful evidence. The aggregated Flemish GP-students and GP-trainers scored worse than their Dutch counterparts. This key finding must be interpreted with care and needs further work. Dutch GP trainees are a few years older than their Flemish counterparts and may have had more clinical experience. Dutch GP trainees tend to work between graduation and starting vocational training.

In the present study, the Flemish students only had a brief preparation period of more or less a month and we have no data as to whether they checked the available guidelines. Furthermore, the presentation of a test format they are not used to, the differences in the GP-training program, and sometimes, typical Dutch idioms, could explain their results. Finally, we only applied one knowledge test and this hampers interpretation of the observed differences between Dutch and Belgium (Flanders) participants.

Individual scores cannot be used for high stake decisions like pass or fail for the Flemish participants. One finding, however, suggests construct validity for examination purposes for the test in Flanders. In one University (University Two) in year 1 trainees were urged to study the abstracts of the National guidelines and subsequently they could earn a credit. Their scores were significantly higher than the scores of other Flemish students. Their scores were significantly better than the other Flemish students of that year. This finding reassures us to some extend that the content of the test covers the guidelines and that actively learning these does improve the score. However, this subgroup only consisted of 23 students and therefore this finding should be interpreted carefully. To explore this further, we should look at concurrent validity with other existing test formats in Flanders, such as the knowledge test of Flanders, the Objective Clinical Examination and performance indicators at the training practices.

In the Netherlands we used routine data that were collected in a real examination context. In Belgium (Flanders) we relied on voluntary participants. This explains the lower share of participants per sub group that participated in Belgium (Flanders). Furthermore, we do not know if the scores of these participants are representative for their group.

### Educational benefits

The educational benefits of collaborative assessment are potentially huge. Firstly, if countries with comparable health systems and scope of general practice collaborate it would increase efficiency of assessment procedures. Costs of data banking and analysis, quality and quantity of production of test items could be considered a valuable asset for participating training centers. This can help to overcome the challenge of renewing items and prevent the item banks becoming outdated.

Secondly, test results can be used to assess progress of trainees in general practice. Kramer et all were able tp show increase of provinciency during their training period using a written test to assess knowledge [22,23]. There is already a body of evidence in the undergraduate curriculum underpinning the formative and summative use of knowledge tests over time [3]. Norman et all argue that feedback on performance steers learning behavior at the undergraduate level [24]. Evidence of longitudinal educational effects of international use in post-graduate training, however, is non existent at present.

Thirdly, international benchmarking of educational programs using aggregated group scores is an interesting avenue. Between medical schools in the undergraduate curriculum this was shown to be feasible. Benchmarking can inform course designers to improve their programs [25]. For this, the test formats need to be well accepted by course designers, teachers and students alike. Future work can establish the common ground if countries with different health systems collaborate. The example of the technical specialty of radiotherapy can be of guidance [11].

In this study, the costs for the donor country encompass about 4 full time equivalent (FTE) faculty time and 1,5 FTE technical assistance. Up to present, the initial costs for Flanders were implicit. Only one part-time junior researcher worked part time on the project for 6 months with some academic supervision. If the project is prolonged, the Flemish Inter-university consortium needs to put more effort into item construction and this would lead to more academic costs. It can be expected that this will be very much less than setting up a high quality Flemish country-specific system.

## Conclusion

This study shows that it is feasible to use a Dutch knowledge multiple-choice test in a neighboring country. Collaboration between countries could increase quality of assessment tests and lead to international integration of the discipline.

More work is needed to see if individual scores of a test from another country can be used for high stake decisions. If countries collaborate in the area of assessing GPs trainees, there is an economical benefit due to increased efficiency. It would lead to greater international integration of the discipline and provide an opportunity for benchmarking and quality improvement of training.

## Competing interests

The authors report no conflict of interest. They were paid by their organisations. They have no financial incentives to gain from this project.

## Authors' contributions

RR, JW and VV are part time GPs and work at the Department. There is no financial relationship with the Dutch collaborating Network. AD was a junior researcher and part-time GP trainee and assigned to this research. HD is on the payroll of the Dutch collaborating Network and is a statistician. All authors read and approved the final manuscript.

## Pre-publication history

The pre-publication history for this paper can be accessed here:

http://www.biomedcentral.com/1471-2296/13/103/prepub
